# Supplementary material for: Integrating Machine Learning and Bulk and Single-Cell RNA Sequencing to Decipher Diverse Cell Death Patterns for Predicting the Prognosis of Neoadjuvant Chemotherapy in Breast Cancer
Source: Int J Mol Sci. 2025 Apr 13;26(8):3682. doi: 10.3390/ijms26083682 (PMC12027272; doi:10.3390/ijms26083682)
Supplement: Supplementary file 1 [file ijms-26-03682-s001.zip › ijms-3521681-supplementary.pdf]

Table S1 Univariate and multivariate logistic regression analysis of factors influencing the efficacy of NAC (including UGCG, age, ER, PR, HER2, and Ki-67 expression).

Characteristic	Univariate Regression					Multivariate Regression				
	β	S.E	Z	*P*	OR (95%CI)	β	S.E	Z	*P*	OR (95%CI)
UGCG										
Low					1.00 (Reference)					1.00 (Reference)
High	1.92	0.85	2.27	0.023*	6.82 (1.30 ~ 35.93)	1.86	0.87	2.15	0.032*	6.43 (1.18 ~ 35.22)
Age										
≤50					1.00 (Reference)					1.00 (Reference)
＞50	-0.25	0.54	-0.45	0.650	0.78 (0.27 ~ 2.26)	-0.23	0.63	-0.37	0.713	0.79 (0.23 ~ 2.72)
ER										
Negative					1.00 (Reference)					1.00 (Reference)
Positive	-1.02	0.91	-1.11	0.265	0.36 (0.06 ~ 2.16)	-0.53	1.16	-0.45	0.649	0.59 (0.06 ~ 5.75)
PR										
Negative					1.00 (Reference)					1.00 (Reference)
Positive	-0.58	0.82	-0.70	0.481	0.56 (0.11 ~ 2.79)	-0.58	1.08	-0.54	0.589	0.56 (0.07 ~ 4.62)
HER2										
Negative					1.00 (Reference)					1.00 (Reference)
Positive	0.08	0.54	0.16	0.877	1.09 (0.38 ~ 3.15)	0.11	0.60	0.19	0.850	1.12 (0.34 ~ 3.64)
Ki67										
≤20%					1.00 (Reference)					1.00 (Reference)
＞20%	0.96	1.26	0.76	0.445	2.61 (0.22 ~ 30.57)	0.82	1.39	0.59	0.554	2.28 (0.15 ~ 34.88)

OR, Odds Ratio; CI, Confidence Interval; *, $p<0.05$

Table S2 Univariate and multivariate logistic regression analysis of factors influencing the efficacy of NAC (including BTG2, age, ER, PR, HER2, and Ki-67 expression).

Characteristic	Univariate Regression					Multivariate Regression				
	β	S.E	Z	*P*	OR (95%CI)	β	S.E	Z	*P*	OR (95%CI)
BTG2										
Low					1.00 (Reference)					1.00 (Reference)
High	-1.46	0.58	-2.52	0.012*	0.23 (0.07 ~ 0.72)	-1.82	0.69	-2.65	0.008*	0.16 (0.04 ~ 0.62)
Age										
≤50					1.00 (Reference)					1.00 (Reference)
＞50	-0.25	0.54	-0.45	0.650	0.78 (0.27 ~ 2.26)	0.03	0.65	0.05	0.964	1.03 (0.29 ~ 3.66)
ER										
Negative					1.00 (Reference)					1.00 (Reference)
Positive	-1.02	0.91	-1.11	0.265	0.36 (0.06 ~ 2.16)	-1.21	1.14	-1.06	0.288	0.30 (0.03 ~ 2.79)
PR										
Negative					1.00 (Reference)					1.00 (Reference)
Positive	-0.58	0.82	-0.70	0.481	0.56 (0.11 ~ 2.79)	-0.44	1.11	-0.39	0.694	0.65 (0.07 ~ 5.71)
HER2										
Negative					1.00 (Reference)					1.00 (Reference)
Positive	0.08	0.54	0.16	0.877	1.09 (0.38 ~ 3.15)	-0.44	0.66	-0.67	0.505	0.65 (0.18 ~ 2.34)
Ki67										
≤20%					1.00 (Reference)					1.00 (Reference)
＞20%	0.96	1.26	0.76	0.445	2.61 (0.22 ~ 30.57)	1.48	1.43	1.04	0.299	4.40 (0.27 ~ 72.04)

OR, Odds Ratio; CI, Confidence Interval; *, $p<0.05$

Table S3 Univariate and multivariate logistic regression analysis of factors influencing the efficacy of NAC (including TNFRSF21, MYB, age, ER, PR, HER2, and Ki-67 expression).

Characteristic	Univariate Regression					Multivariate Regression				
	β	S.E	Z	*P*	OR (95%CI)	β	S.E	Z	*P*	OR (95%CI)
TNFRSF21										
Negative					1.00 (Reference)					1.00 (Reference)
Positive	1.31	0.59	2.23	0.025*	3.71 (1.18 ~ 11.74)	1.52	0.62	2.45	0.014*	4.57 (1.35 ~ 15.44)
Age										
≤50					1.00 (Reference)					1.00 (Reference)
＞50	-0.25	0.54	-0.45	0.650	0.78 (0.27 ~ 2.26)	-0.16	0.64	-0.25	0.799	0.85 (0.24 ~ 2.96)
ER										
Negative					1.00 (Reference)					1.00 (Reference)
Positive	-1.02	0.91	-1.11	0.265	0.36 (0.06 ~ 2.16)	-1.29	1.14	-1.14	0.256	0.28 (0.03 ~ 2.55)
PR										
Negative					1.00 (Reference)					1.00 (Reference)
Positive	-0.58	0.82	-0.70	0.481	0.56 (0.11 ~ 2.79)	-0.06	1.11	-0.06	0.955	0.94 (0.11 ~ 8.35)
HER2										
Negative					1.00 (Reference)					1.00 (Reference)
Positive	0.08	0.54	0.16	0.877	1.09 (0.38 ~ 3.15)	0.14	0.60	0.24	0.808	1.16 (0.36 ~ 3.72)
Ki67										
≤20%					1.00 (Reference)					1.00 (Reference)
＞20%	0.96	1.26	0.76	0.445	2.61 (0.22 ~ 30.57)	1.63	1.34	1.22	0.224	5.11 (0.37 ~ 70.88)

OR, Odds Ratio; CI, Confidence Interval; *, $p<0.05$

Table S4 Univariate and multivariate logistic regression analysis of factors influencing the efficacy of NAC (including MYB, age, ER, PR, HER2, and Ki-67 expression).

Characteristic	Univariate Regression					Multivariate Regression				
	β	S.E	Z	*P*	OR (95%CI)	β	S.E	Z	*P*	OR (95%CI)
MYB										
Negative					1.00 (Reference)					1.00 (Reference)
Positive	-1.46	0.58	-2.52	0.012*	0.23 (0.07 ~ 0.72)	-1.63	0.62	-2.64	0.008*	0.20 (0.06 ~ 0.66)
Age										
≤50					1.00 (Reference)					1.00 (Reference)
＞50	-0.25	0.54	-0.45	0.650	0.78 (0.27 ~ 2.26)	-0.37	0.65	-0.56	0.572	0.69 (0.19 ~ 2.47)
ER										
Negative					1.00 (Reference)					1.00 (Reference)
Positive	-1.02	0.91	-1.11	0.265	0.36 (0.06 ~ 2.16)	-0.65	1.14	-0.57	0.565	0.52 (0.06 ~ 4.84)
PR										
Negative					1.00 (Reference)					1.00 (Reference)
Positive	-0.58	0.82	-0.70	0.481	0.56 (0.11 ~ 2.79)	-0.31	1.11	-0.28	0.782	0.74 (0.08 ~ 6.47)
HER2										
Negative					1.00 (Reference)					1.00 (Reference)
Positive	0.08	0.54	0.16	0.877	1.09 (0.38 ~ 3.15)	0.34	0.61	0.56	0.573	1.41 (0.43 ~ 4.66)
Ki67										
≤20%					1.00 (Reference)					1.00 (Reference)
＞20%	0.96	1.26	0.76	0.445	2.61 (0.22 ~ 30.57)	1.65	1.34	1.23	0.220	5.19 (0.37 ~ 72.07)

OR, Odds Ratio; CI, Confidence Interval; *, $p<0.05$
